# Strong Inference for Systems Biology

**DOI:** 10.1371/journal.pcbi.1000459

**Published:** 2009-08-28

**Authors:** Daniel A. Beard, Martin J. Kushmerick

**Affiliations:** 1 Biotechnology and Bioengineering Center, Department of Physiology, Medical College of Wisconsin, Milwaukee, Wisconsin, United States of America; 2 Department of Radiology, University of Washington, Seattle, Washington, United States of America; University of California, San Diego

## Introduction

Platt's 1964 essay on strong inference [1] illuminates a rational approach to scientific inquiry that integrates seamlessly with current investigations on the operation of complex biological systems. Yet in re-examining the 1964 essay in light of current trends, it is apparent that the groundbreaking approach has failed to become universal. Here it is argued that both the opportunity and the need to follow Platt's advice are now greater than ever. A revised method of strong inference for systems biology is presented and applied to analyze longstanding questions in cardiac energy metabolism. It is shown how this logical framework combined with computational-based hypothesis testing illuminates unresolved questions regarding how the energetic state of the heart is maintained in response to changes in the rate of ATP hydrolysis.

## Philosophical Framework

### Platt's Strong Inference

A half century ago Platt proposed a formal schema for scientific inquiry based, in part, on his assessment of the rapid progress made in molecular biology and theoretical physics in the middle part of the twentieth century. Building on the “simple old-fashioned method of inductive inference that goes back to Francis Bacon” [1], Platt proposed a method of strong inference, applied through the following logical sequence:

“1. Devising alternative hypotheses;

2. Devising a crucial experiment (or several of them), with alternative possible outcomes, each of which will, as nearly as possible, exclude one or more of the hypotheses;

3. Carrying out the experiment so as to get a clean result;

1′. Recycling the procedure, making subhypotheses or sequential hypotheses to refine the possibilities that remain; and so on.”

Current use of the scientific method ideally applies this logic, but all too often work stagnates at a particular step without completing the cycle. In adopting and effectively applying Platt's steps, hypotheses are systematically disproved and refined, successively moving toward a more complete understanding of nature. Hypotheses are not precious “personal property” to be protected and defended. Instead, successful disproof is the key to progress. Likewise, the goal of publication of an experimental result is to alert the community of progress and invite criticism, alternative explanations, and—what often hurts an investigator's ego—a more rigorous experiment.

Platt warns that when alternative hypotheses are not sought, scientific inquiry becomes a “conflict between men, each with his single Ruling Theory.” In that case, “the scientist has no choice but to be either soft-headed or disputatious.” (Where unattributed, all quotations are from Platt [1].) Such situations remain too familiar in science. Indeed today's reader might suggest that Platt's diagnosis applies as well today as it did forty years ago. In other words, Platt did not entirely succeed in establishing strong inference as the standard operating procedure for science. Here, we argue that if the sickness that Platt identified remains, we can still move toward a cure by adopting, and perhaps recalibrating, strong inference for application in systems biology. The opportunity exists to perhaps realize Platt's proposed methodology on a scale greater than he may have imagined.

What are the symptoms of the sickness that Platt identified? Platt enumerates several: The Frozen Method, The Eternal Surveyor, The Never Finished, The Great Man with a Single Hypothesis, The Little Club of Dependents, The Vendetta, The All-Encompassing Theory Which Can Never Be Falsified. It may be that a major emphasis on technology development has alleviated the problem of frozen methodology. It is probably fair to say that biology has become even more method-oriented than in Platt's day. Regarding the other symptoms, we suspect that most readers can quickly identify current examples within their own fields of specialization.

### Systems Biology

While many of these symptoms remain, it is also clear that several revolutionary developments have shaped the scientific landscape since the 1964 publication of Platt's essay. One of the most obvious and critical was the development and application of genome sequencing technology, highlighted by the Human Genome Project. Now in the era of post-genomics and systems biology, it is widely appreciated that biology is largely discovery-driven and perhaps less hypothesis-driven than in the past. As has been convincingly argued, discovery-driven and hypothesis-driven research are really not fundamentally distinct, so long as careful logic is applied in cycling between generating ideas and data to test the ideas [2]. It is (or at least should be) only a question of whether one starts at Step 1, 2, or 3 in Platt's cycle. Where one starts depends on the tools at one's disposal and the prior knowledge available. Regardless, after a few iterations progress will be neither uniquely discovery-driven nor hypothesis-driven. If large-scale data collection happens to be a good place to start tackling a particular problem, it is not a good place to end. Hypothesis-generating (discovery-driven) experiments are fine, as long as actual hypotheses are generated, tested, disproved, refined, etc.

However, while discovery-driven approaches to research should offer no philosophical resistance to strong inference, in practice they have. The problem was foreshadowed by Platt who cautioned that we not “become method-oriented rather than problem-oriented.” In the twenty-first century biology has become unduly method-oriented; new tools to generate data appear to be the most highly prized. Perhaps this trend originated in the Human Genome Project. A 2000 White House press release announced the completion of the first draft sequence of the human genome, promising “a new era of molecular medicine” in which scientists will (among other things) “discover which DNA sequence changes in a gene can cause disease” and “develop new treatments at the molecular level.” Surely the project was an enormous success, providing not just the sequence itself, but also new technologies for sequencing and associated tasks that have become indispensible parts of the biomedical research toolbox. Yet nearly a decade and a half later, progress on many of the health concerns described in the White House press release proceeds much as it did before, during, and immediately after completion of the Human Genome Project. Thanks in part to information revealed by the Human Genome Project, it is now appreciated that most diseases (including diabetes, cancer, and Parkinson disease, which are specifically mentioned in that particular press release) involve a complex interplay between many genes and environmental factors. Sequences and sequencing technologies are helping us to see where there are gaps in knowledge and helping to fill in those gaps. The sequence itself neither identifies the gaps nor fills them in.

The point is that large-scale sequencing has introduced important new tools—perhaps even revolutionary tools—for biomedical research, while the underlying logical framework for “exploring the unknown” has remained unchanged. Some hype and oversimplification might be expected in promoting a grand technological achievement. But the Human Genome Project truly set a new standard for expectations. With this achievement hailed as an epoch-changing event, what's next? If the epoch has changed, what does that mean for the old-fashioned scientist who is still engaged in careful, cautious hypothesis testing? Why would anyone want to make deliberate progress on a particular biological question when the lab down the hall is getting the attention (and funding) for fully embracing The Next Really Big Thing?

Systems biology is one of the names we are calling the next big thing these days. More than just a name, systems biology represents a potential vehicle for systematic application of Platt's principles to biomedical research. The opportunity exists because the need is especially obvious at this particular time. Former NIH director Elias Zerhouni points out that while “discovery in the life sciences is accelerating at an unprecedented rate,” we are now faced with the critical “need to understand complex biological systems” [3]. The now familiar paradigm is that the pace of data generation requires the development of new tools to systematize and synthesize results and to simulate complex biological systems.

### Computational Biology

Apparently Platt did not hold much stock in mathematical modeling and simulation: “Equations and measurements are useful when and only when they are related to proof: but proof or disproof comes first and is in fact strongest when it is absolutely convincing without any quantitative measurement.” This is perhaps where Platt's vision needs to be recalibrated for the times. The mechanisms underlying the operation of biological systems—e.g., gene regulatory networks, metabolic networks, signaling networks, and the interoperation of all of the above—cannot be cast in a meaningful way into the simple qualitative framework of an earlier era of molecular biology. Increasingly, describing the operation of the biological systems under current investigation requires invoking computational models to simulate the system. For example, efforts to capture the physiome and construct a virtual human simulation represent the height of ambition of computational biology [4],[5]. Whatever Platt thought of quantitative methods, computational and systems biology will achieve optimal progress by keeping in mind that “the mathematical box is a beautiful way of wrapping up a problem, but it will not hold the phenomena unless they have been caught in a logical box to begin with.”

The fundamental key is to recognize that a computational model, however simple or complex, is a hypothesis, or perhaps a series of hypotheses bundled together into a set of equations or a computer algorithm that best represents how we think a complex systems works. Formulating hypotheses as computational models has at least two advantages when using strong inference to systematically uncover the important mechanisms governing a complex system:

1. Computational/mathematical models are precise. Cartoons and thought models constructed out of words serve useful purposes in textbooks and in discussion sections of articles. However, when qualitative methods (such as drawing boxes and arrows to indicate interactions in a molecular network) are used to describe a hypothesis, that hypothesis can be interpreted many ways, and is therefore difficult to disprove without ambiguity.
2. Computational/mathematical models, as necessarily simplified representations of reality, are sometimes useful but always incomplete. By virtue of their incompleteness, computational models fit naturally into Platt's scheme. Models can remain the precious “personal property” of their inventors and still be subject to disproof and refinement. It is the natural process of mathematical and computational modeling to close the loop between Platt's Step 3 and Step 1.

Closing the loop, of course, requires data. After all, “without data, there is nothing to model; and without models, there is no source of deep predictive understanding” [6]. Thus, the modeling and the data collection efforts must be tightly integrated. To properly invoke strong inference in systems biology, models must be formulated as formal precise hypotheses to be disproved and refined in light of relevant data.

## Applications in Cardiac Energetics

### Overview

In the remainder of this essay we apply strong inference to analyze two long-standing questions in the control and regulation of cardiac energy metabolism: the metabolic stability hypothesis—the hypothesis that the rate of oxidative ATP synthesis in the heart is not influenced by any significant changes in substrate concentrations with changes in rate of ATP hydrolysis—and the phosphocreatine shuttle theory—a loosely organized set of ideas regarding possible roles of creatine and phosphocreatine in transporting ATP hydrolysis potential in the cardiomyocyte. These examples show how significant progress can be made on largely stalled areas of investigation by applying strong inference.

### Example 1: Metabolic Stability in the Heart

#### The biological question

**Figure 1:**
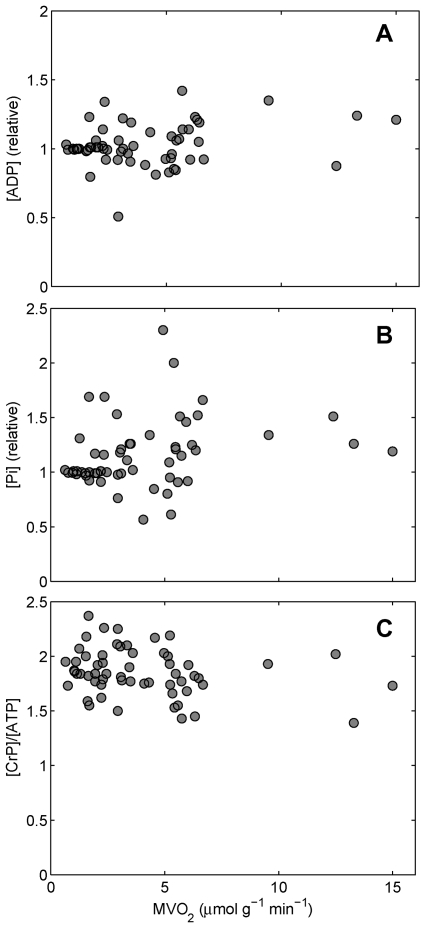
Relative (normalized to baseline) cardiac [ADP], [Pi], and [CrP]/[ATP] versus cardiac oxygen consumption rate (MVO_2_) from Katz et al. [9]. (A) [ADP], (B) [Pi], (C) [CrP]/[ATP]. Data were obtained from ^31^P-MRS of the canine myocardium, with MVO_2_ varied by pacing or infusion of epinephrine or phenylephrine. Data are adapted from Figures 2, 4, and 6 of Katz et al. [9].

The landmark metabolic stability hypothesis was formulated based on the observation that the heart maintains relatively stable concentrations of phosphate metabolites over the observed range of cardiac oxygen consumption. This phenomenon was first reported by Neely et al. [7], who measured phosphocreatine (CrP), ATP, ADP, and AMP in rapidly frozen samples from a Langendorff isolated heart preparation. They showed that when cardiac work was increased by increasing aortic pressure, the concentrations of these metabolites remained remarkably constant. The first in vivo observations of this phenomenon were reported by Balaban et al. [8], who reported similar results on ATP and CrP based on ^31^phosphate magnetic resonance spectroscopy (^31^P-MRS) of the canine myocardium. That study was followed up by Katz et al. [9] with measurements of the CrP/ATP ratio and inorganic phosphate concentration and estimates of ADP as functions of myocardial oxygen consumption rate (MVO_2_). The data from the Katz et al. study are reproduced here in Figure 1, in which data from three different protocols used to increase MVO_2_ (pacing, epinephrine, and phenylephrine infusions) are lumped together in one set of figures for ADP (relative to baseline), inorganic phosphate (Pi, relative to baseline), and CrP/ATP ratio.

Although the experiments summarized in Figure 1 were conducted twenty years ago, they exemplify the process of making observations and generating hypotheses to explain what is observed. The study combined “discovery science,” because new tools were used to make novel observations, with “hypothesis-driven science,” because a specific hypothesis was generated. Since no trend of increase or decrease of CrP, Pi, or ATP with increased MVO_2_ is discernable from the data, it was concluded that these variables are not responsible for modulating the rate of oxidative ATP synthesis in vivo, and the hypothesis of feedback regulation by ADP and Pi was declared disproven.

The hypothesis that the substrates for oxidative phosphorylation (ADP and Pi) are not the primary regulators of oxidative phosphorylation has been stated by Balaban:

“…recent data from intact tissues with high oxidative phosphorylation capacities (i.e., heart, brain, and kidney) indicate that the cytosolic concentration of ADP and Pi do not change significantly with work. These data imply that [a] simple feedback model is not adequate to explain the regulation of energy metabolism in these tissues…” [10].

The insufficiency of feedback regulation has inspired a number of theories to explain the control of oxidative phosphorylation in the heart in vivo. Indeed, several putative explanations of metabolic stability in the heart have been formulated as computational models simulating cardiac energy metabolism. Examples include: feed-forward activation by Ca^2+^, in which Ca^2+^ ion stimulates oxidative phosphorylation mainly through activating tricarboxylic acid (TCA) cycle enzymes [11]; and the phosphocreatine shuttle system, in which ATP is supplied to local sites of ATP hydrolysis in the cell by rapidly diffusing phosphocreatine, while ATP and/or ADP diffusion is severely restricted [12]. These proposed explanations for cardiac metabolic stability represent state-of-the-art theoretical work in the field. In addition, Korzeniewski [13] has promoted the theory, termed “parallel activation”, that the activities of the enzymes and transporters involved in ATP synthesis change in synchrony with and proportional to changes in the rate of ATP utilization. The parallel-activation theory is thus a higher-level abstraction that is not necessarily in conflict with the proposed biochemical mechanisms explaining metabolic stability in the heart.

Yet, however important it has been in inspiring theory and experiments, the above quotation laying out the hypothesis of metabolic stability was not authored under the philosophy of strong inference. In fact, while we have recast the message as a hypothesis, this and similar statements are presented as conclusions in the original sources. The metabolic stability hypothesis is widely accepted and serious effort has been put into trying to explain it. Yet if the hypothesis is disprovable, then the longer it goes un-disproved, the less it inspires and the more it impedes progress.

#### An alternative dataset leads to an alternative hypothesis

**Figure 2:**
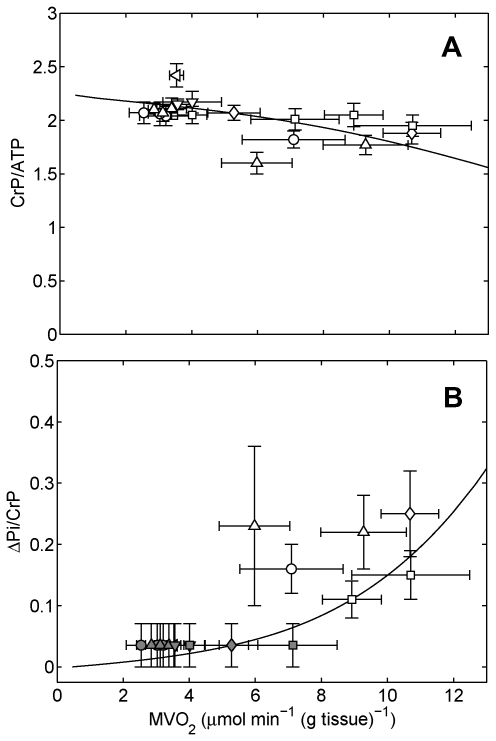
Steady-state phosphate metabolites in the canine heart measured by ^31^P-MRS. (A) CrP/ATP is plotted versus cardiac oxygen consumption rate (MVO_2_). (B) ΔPi/CrP is plotted as a function of MVO_2_. Solid lines indicate model predictions from [31]; data are adapted from: ○, Zhang et al. [14]; ◃, Zhang et al. [15]; ⋄, Gong et al. [16]; ▵, Ochiai et al. [17]; ▿, Gong et al. [18]; □, Bache et al. [19]. This figure is adapted from Wu et al. [31], with permission. Shaded data points indicate situation when Pi is below limit of detection.

Figure 2 reproduces in vivo ^31^P-MRS data on cardiac phosphate metabolites reported in a series of studies by Zhang and coworkers. Here we see that the trend of relatively stable CrP/ATP versus MVO_2_ observed by Katz et al. [9] is reproduced by Zhang et al. [14]–[19], while some differences in the observations are apparent. First, we note that ADP data are not reported in Figure 2, because ADP is not directly detectable by ^31^P-MRS in vivo. The data points in Figure 1A are computed from the expression for creatine kinase (CK) equilibrium, assuming that measured ATP signal indicates relative cytoplasmic ATP concentration, with no significant impact from changes in mitochondrial ATP. Whether or not that model is reasonable, the ADP data in Figure 1A represent a derived variable, and not a set of independent measurements for comparison to theoretical predictions.

Also note that in Figure 2B the data on Pi at low MVO_2_ do not correspond to direct measurements. This is because using newer MRS technology that provides a more spatially localized signal than was possible in the Katz et al. study, it has been repeatedly demonstrated that Pi is not detectable in the myocardium at baseline. Since the baseline Pi is not measured, Pi is appropriately reported as ΔPi, the measured increase over baseline (here normalized to the CrP signal). The shaded data points in Figure 2B correspond to the cases where Pi falls below the limit of detection, which is approximately 1 mM [19]. The range of ΔPi/CrP indicated by the error bars for those data points corresponds to the range of zero to the limit of detection.

It is possible that the baseline signal identified as Pi by Katz et al. [9] was due to 2,3-diphosphoglycerate in the blood pool of the left ventricle cavity. Regardless, in the 20 years since publication, the Pi data of Katz et al. have not been unambiguously reproduced in the literature. A recent review [20] cites ten studies demonstrating “that during increases in work demand the levels of ATP, CrP, and other energy metabolites do not change even though the flux through these pools changes many fold” [7]–[9], [21]–[27]. However, with the exception of the 1989 Katz et al. study and the 1990 study by Detre et al. [26], none of these studies report measurements of Pi in the heart in vivo as a function of cardiac work. Detre et al. report that “No changes in high-energy phosphates were observed except at the highest rate-pressure products obtained, where small increases in inorganic phosphate and decreases in the phosphocreatine/ATP ratio were observed.” Since neither relative nor absolute changes in Pi concentrations were reported quantitatively, the use of the qualifier “small” by Detre et al. is unfortunately vague.

Contrasting that evidence for constant Pi with, for example, the six studies summarized in Figure 2B which all show a statistically significant increase in measured in vivo Pi with MVO_2_, the case for constant Pi is not strong. Leaving the derived variable ADP out of our analysis, what is left from Figure 1 to focus on are the data on CrP/ATP versus MVO_2_. While a line of zero slope passes through this data cloud, these data certainly do not prove that the relationship underlying these data is a line of zero slope. A line of zero slope becomes a slightly less convincing description when compared to the data of Figure 2A, which are more consistent with CrP/ATP decreasing from approximately 2.1 at baseline to 1.8 at maximally stimulated cardiac work. Furthermore, since the data in Figure 2A are consistent with the data of Figure 1C, we can count that aspect of the groundbreaking study of Katz et al. [9] successfully reproduced, both by studies cited here and by many others.

Synthesizing the available data on phosphate metabolites in the normal heart in vivo shows: CrP/ATP decreases either slightly (perhaps 15%) or not at all with increases in MVO_2_, while Pi increases from somewhere below the minimum detectable concentration at baseline to more than two times the minimum detectable concentration at maximal or near maximal MVO_2_. Even in the detectable range the Pi measurements suffer from substantial noise, resulting in large uncertainty in ΔPi/CrP. Thus the nature of the relationship between Pi and MVO_2_ is not clearly revealed due to noise and individual variability in the measurements. Moreover, due to both the low signal-to-noise ratio and the fact that the Pi signal originates from both the cytoplasm and the mitochondrial matrix, absolute Pi concentrations (in mass per unit volume) are not easily estimated from these relative measurements.

These data inspire the formulation of an alternative to the metabolic stability hypothesis. Formally, we state the metabolic stability hypothesis as “biochemical feedback is not adequate to explain the regulation of energy metabolism in the heart,” and its alternative, “biochemical feedback is adequate to explain the regulation of energy metabolism in the heart.”

#### Testing the hypotheses using computer simulations

These alternative hypotheses are clearly mutually exclusive, satisfying the requirement of Platt's Step 2. In this case, since these hypotheses relate to potential mechanisms underlying data that have already been collected, Platt's Step 3 takes the form of a simulation experiment. Specifically, if an independently validated simulation of energy metabolism in the heart, in which the rate of oxidative phosphorylation is controlled by biochemical feedback, is able to match the available data, then the stability hypothesis will be disproved and the alternative proved. (Formal proof of the stability hypothesis would be difficult since it would require exhaustively ruling out all theoretical possibilities. Luckily, we shall disprove the stability hypothesis, demonstrating that such a proof is impossible.)

In a recent series of papers we have developed a computer model of cardiac energy metabolism that provides the means to distinguish the hypotheses. Briefly, the model to simulate mitochondrial metabolism and electrophysiology is based on a large number of kinetic and steady-state data from purified mitochondria [28]–[30]. The cellular region is subdivided into cytoplasmic and mitochondrial compartments. The mitochondrial metabolism model incorporates TCA cycle fluxes, mitochondrial oxidative phosphorylation fluxes, and substrate and cation transport fluxes. The cytosolic region includes the adenylate kinase and CK reactions (which are assumed to operate near equilibrium) and an ATP hydrolysis reaction that represents ATP-consuming processes in the cell. Thus the model simulates oxidative ATP synthesis in a feedback controlled, demand-driven manner. As the rate of ATP hydrolysis increases, products of ATP hydrolysis (which are substrates for ATP synthesis) build up. An increase in substrates for synthesis stimulates an increase in the rate of synthesis.

Integrating this energy metabolism model with a model to simulate oxygen transport in the myocardium, we are able to simulate the experiments summarized in Figures 1 and 2. Model predictions, which are plotted as solid lines in Figure 2, directly contradict the metabolic stability hypothesis. Specifically, our model, which invokes no mechanisms for control of oxidative phosphorylation other than the simple feedback mechanism described above, is able to adequately explain the regulation of energy metabolism in the heart [31]. In this case, computer simulation helped, as J. E. Bailey put it, “to think (and calculate) logically about what components and interactions are important in a complex system” [32] in order to confidently challenge a firmly established conclusion/hypothesis.

#### Thawing out a frozen question

Furthermore, these simulations help resolve an old scientific debate that was never adequately concluded. One of us recalls that twenty years ago the findings of Balaban and colleagues generated extensive and compelling discussions both in the literature and at scientific meetings: “You did not consider alternative hypotheses!”, “The methods are not precise enough to justify your conclusion!”, “How did you calibrate the NMR [nuclear magnetic resonance] spectral signals into concentrations?”, “How do you know that the CK is maintained at equilibrium in the cell so that the ADP concentration can be calculated?”, “What experiment can distinguish feed-back from feed-forward control?” While such questions were debated extensively at poster sessions for years, the critical disproofs that came later were presented in terms that decidedly avoided challenging the status quo.

Recall that the metabolic stability hypothesis is formulated on the foundation that both biochemical substrates for oxidative phosphorylation, ADP and Pi, remain essentially constant in the heart. Thus the observations of significant increases in Pi with MVO_2_ are appropriately interpreted as a simple direct disproof. Yet, those observations were presented as consistent with the Balaban data. Specifically, much was made of the fact that the later experiments achieved rate-pressure products (RPP) significantly higher than the earlier studies and that only at the higher values of RPP does the Pi signal become observable. Making this point, one of us is guilty of this sort of equivocation intended to avoid conflict. In discussing our model predictions in comparison to the data on inorganic phosphate, Wu et al. [31] state, “Our model predictions agree with [the observations of Katz et al. and Zhang et al.] that the total Pi (cytosolic plus mitochondrial) remains constant within limits of detection at moderate work rates.” Such statements are crafted to ensure that all parties remain emotionally satisfied, but avoid recognizing several large elephants sitting in the room: First, the phrase “constant within limits of detection” says nothing more than “there is no conflict in the regime where there are no data.” Second, in the regime where there are data, Pi does change significantly with MVO_2_. So the emphasized point of agreement is trivial while the crucial point of disagreement is deemphasized.

Putting equivocating statements aside, the experiments of Zhang and colleagues directly contradict the assertion that concentrations of substrates for ATP synthesis do not significantly change in response to changes in ATP hydrolysis rate. By comparing computer simulations to these data, we are able to firmly disprove the associated metabolic stability hypothesis. This outcome is an affirmation of strong inference. The stability hypothesis of Balaban and coworkers has stood for twenty years and inspired deeper investigations into the control of energy metabolism in the heart. Its disproof is an opportunity for further refinement and progress. Furthermore, this disproof does not represent a proof of our computational model. However, the integrated model has been validated on the basis of a comparison of the data in Figure 2 and additional independent experiments described in Wu et al. [31],[33]. Having survived our attempts at disproof, the model may provide a useful theoretical tool to update and refine established ideas about energy metabolism in the heart. While the current model has survived initial tests, more experiments and deeper analyses will undoubtedly lead to refinement and perhaps outright disproof. Useful challenges will adopt the strong inference/systems biology cycle as the logical plan of attack.

### Example 2: The Phosphocreatine Shuttle

#### The biological question

**Figure 3:**
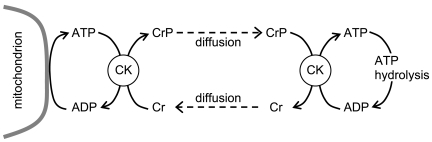
Facilitated diffusion of ATP hydrolysis potential by the creatine kinase system. Phosphocreatine (CrP) is synthesized from ATP near sites of ATP synthesis and diffuses to sites of ATP hydrolysis, where ATP is synthesized from CrP.

Histological inspection of a skeletal and cardiac myocyte shows that they do not resemble a reaction chamber akin to a well-stirred chemistry flask. Bessman and Geiger [34] developed a notion of intracellular transport of metabolites based on localization of enzymes and isoforms of enzymes. They argued for limited diffusion of ATP and ADP and for a flux circuit of PCr and Cr between sites of ATP use and generation that they called “the creatine phosphate shuttle.” Figure 3 depicts the circuit envisioned wherein ATP and ADP diffusion between compartments is negligible. Hochachka generalized these ideas [35] and used them to account for the problem already discussed in Example 1 above, namely how is flux in an metabolic pathway regulated over a wide range (≫2-fold) when substrates within the cell are close to their apparent *K*_m_ values and do not change appreciably with flux? Hochachka showed how intracellular microcompartments could account for “metabolic stability.” The laboratories of Walliman (e.g., [36]) and Saks (e.g., [37]–[39]) have introduced many additional experimental findings and conceptual developments on these same questions and added much detail on the transport of adenine nucleotides coupled to creatine and creatine phosphate exchange in the mitochondria.

Hypotheses about the nature of the cellular interior and the properties of water as solvent and metabolites as solutes remain fruitful avenues of investigation. Macroscopic features can hide microscopic properties. For example, measured diffusivities of water and of low molecular weight solutes in gels that are macroscopically a solid, such as a gelatin cube, are slightly reduced compared to diffusivity in pure aqueous solution. From these studies Wang developed a view of proteins as macromolecular strands in an aqueous environment that obstruct random three-dimensional diffusion and so decrease the measured diffusivity by a calculable extent [40]. Myofibrils, sarcoplasmic reticulum, and mitochondria are organized in a regular crystal-like pattern [41],[42]; ions, such as Ca^2+^, and metabolic reactants diffuse between intracellular compartments and amongst large intracellular proteins. The spatial organization is important, for example, in Ca^2+^ signaling in the heart, in which ion fluxes establish and depend on spatial gradients in the cytosol. Early diffusion studies in skeletal muscle fibers showed that the relatively low concentration of Ca^2+^ combined with the high concentration and the affinity of binding sites for the ion cause apparent diffusivity of total (free plus bound) Ca^2+^ to be orders of magnitude lower than other charged and uncharged solutes [43]. Although the simulation of cardiac energy metabolism described in Example 1 accounted for a range of experimental data, it did not explicitly account for diffusive transport of energy metabolites within and around micrometer-scale intracellular structures, including myofibrils, sarcoplasmic reticulum, and mitochondria. As such, the success of those simulations argues against the need for more complex features in the cytoplasm. Yet, it has been proposed that significant concentration gradients occur between the myofibrils and other structures, and the mitochondria and across the mitochondrial membranes, and that a “phosphotransfer network,” modulated through special localization of different CK isoforms, is critical to the regulation of oxidative phosphorylation in vivo [37],[44]. The latter work described the “phosphocreatine shuttle” and generated large cytosolic gradients of ADP provided a large permeability barrier was included; their factor *r* in the diffusion equations was responsible for the enhanced gradients. These contrasting views of metabolite localization and mobility are mutually exclusive.

We now consider how strong inference can advance these questions. Two issues are intertwined and need to be separated. First, the descriptions of “the creatine phosphate shuttle” and “intracellular compartments” admit various definitions and interpretations; compartments bounded by membranes are clearly defined. The concepts of the shuttle and compartmentalization have been described qualitatively from the original diagrams in [34] to the more detailed ones in [36]. However, except for [37],[44] and [45], most of the discussion in the literature has been qualitative; alternative hypotheses need to be clearly stated with quantitative definitions and the possibility of being falsified by experiments in intact cells to satisfy the criteria of Platt's strong inference.

#### The facilitated diffusion theory

Meyer et al. [45] analyzed this system by considering two extreme theoretical possibilities based on those originally posed by Bessman and Geiger [34]; see Figure 1 in [45] for descriptions of each. The two hypotheses were the obligatory shuttle (Figure 3 in [34]) and an ordinary reaction-diffusion scheme termed the facilitated diffusion shuttle hypotheses. Both hypotheses were formulated as computational models where the myofibrillar space was treated as a spatially distributed reaction-diffusion system. In the obligatory shuttle there is no diffusive flux of ATP or ADP between the site of ATP demand (myofibrils) and ATP source (mitochondria); all the flux is conducted by CrP and Cr transport. This hypothesis requires that the spatially averaged CK flux is determined by the magnitude of the ATP hydrolysis flux averaged over the entire cell volume and therefore changes proportionately with ATP flux. In the facilitated diffusion shuttle, ATP, ADP, CrP, Cr, and Pi all diffuse homogeneously, although with potentially different effective molecular diffusivities. By calculating the relative contributions of CrP/Cr diffusion versus ATP/ADP diffusion, it was concluded that when the ratio of CrP+Cr to ATP+ADP was high, the energy flux was carried mainly by CrP/Cr [45] even though only random diffusion mechanisms were considered. The energy flux is borne by those molecules that are present in greatest number, and the total energy flux is the sum of the two parallel fluxes. Furthermore, using measured diffusivities and enzyme activities, it was shown that the facilitated diffusion hypothesis is consistent with the diffusing metabolites ATP, ADP, CrP, and Cr maintained locally at the mass-action ratio of equilibrium of the CK reaction. (The term “facilitated diffusion” describes the parallel diffusion of ATP and PCr so that the total energy transport is facilitated.)

In experiments made specifically to falsify the “obligatory shuttle” or the “facilitated shuttle” hypotheses, NMR polarization transfer methods were used to measure unidirectional fluxes of the CK reaction: CrP→ATP and the reverse [46]. This muscle contains a uniform cell type in which the intracellular distance between myofibrils and mitochondria is larger than in the cardiomyocyte, making a test of the “obligatory shuttle” hypothesis more stringent than in cardiac muscle. The shuttle hypothesis requires the CK flux to be proportional to the ATP flux; the alternative has no such requirement, and both fluxes are determined by the properties of the enzymes and the concentration of their substrates and products. The essential results were that over a 10-fold range of ATPase flux, CK fluxes were nearly equal in both directions, i.e., near equilibrium as predicted by ordinary diffusion theory of randomly mixed substrates and products. Furthermore, the magnitude of the CK flux was several-fold higher than the ATP hydrolysis flux, and thus high enough for the CK reaction to buffer ATP hydrolysis potential. In addition, the absolute value of the polarization transfer flux was approximately equal to predictions from the facilitated diffusion hypothesis (given CK activity measured in extracts of the muscle and accounting for the facts that *V*_max_ in vitro is determined without reverse flux from products and without substantial binding of both products or both reactants) and not consistent with predictions from the obligatory shuttle hypothesis. Thus these experiments apply Platt's strong inference by disproving the obligatory shuttle hypothesis, at least for certain skeletal muscle tissues, and not disproving the facilitated diffusion model.

(Walliman [47] criticized these experiments on several grounds, one of which is that global measurements cannot address the existence and function of localized enzymes in compartments. It is certainly true that the NMR measurements detect the global averages. Many further experiments from Wallimann's lab demonstrated localization of CK to myofibrils, inner mitochondrial space and membrane, Sarca/Endoplasmic Reticulum Ca^2+^-ATPase (SERCA), and elsewhere on muscle structures. Enzyme localization does not mean the metabolites are similarly localized. Because of high diffusivity and concentration, the local concentrations of CrP, Cr, and ATP exceed, by at least an order of magnitude, the binding constants of the enzyme, as discussed by Meyer et al. [45]. A second criticism is that NMR measures only the “visible” metabolites, and may not include those in certain compartments. But earlier experiments both in cardiac and skeletal muscle [48] showed that neutralized perchloric acid extracts made from these organs had the same concentrations and metabolite ratios as those in the spectra of intact tissues. Thus there is no significant fraction of metabolites not detected by conventional NMR techniques, with the caveat that experimental error is about 10%.)

The phosphocreatine shuttle hypothesis assigns a transport role to the CK system that is more significant and complex than the facilitated diffusion process described by Meyer et al. [45]. Implicit in Platt's scheme is the requirement that hypotheses are stated clearly and concretely so that they may be subjected to unambiguous attempts at disproof. Although a vast literature exists on the subject, few concrete quantifiable statements defining the phosphocreatine shuttle can be found in agreement with the philosophy of strong inference. One can assemble (somewhat subjectively) a set of the consistent themes that distinguish the literature that invokes and supports the phosphocreatine shuttle hypothesis from the literature that does not. Doing so, one finds three major components of the hypothesis: (1) The diffusivity of adenine nucleotides between mitochondria and myofibrils is selective and severely restricted; (2) there exists functional coupling—direct product/substrate channeling—between adenine nucleotide translocase (ANT) and the mitochondrial CK enzyme; and (3) the CK reaction is significantly shifted away from equilibrium in certain intracellular compartments. These three subhypotheses of the phosphocreatine shuttle distinguish the phosphocreatine shuttle hypothesis from the conceptually and mechanistically simpler roles of the CK system as temporal and spatial buffers for the ATP hydrolysis potential. Each hypothesis can be falsified because it is stated quantitatively and has an alternative.

#### Subhypothesis 1: Diffusion of adenine nucleotides is restricted; the alternative is not restricted

The facilitated diffusion theory predicts that there are “no significant diffusion gradients” of ATP, ADP, or Pi over the distance from mitochondrion to local sites of ATP hydrolysis in the normal cardiomyocyte [45], and that even in the absence of CK, none of these concentrations vary spatially by more than 2% in steady state. Thus, the facilitated diffusion theory predicts that although CrP is a major carrier of the ATP hydrolysis potential when CK is active, the CrP shuttle is not essential to maintain steady-state concentrations of energy metabolites in the cardiomyocyte. In fact, the regular and approximately hexagonal packing of mitochondria in the plane perpendicular to the major axis of the cell [42] would serve to minimize diffusion distances and associated gradients of phosphate metabolites.

The hypothesis that there is no significant diffusion barrier between sites of mitochondrial ATP synthesis and cellular ATP hydrolysis in the cardiomyocyte is supported by a set of experiments reported by Kaasik et al. [49], in which contractile kinetics were assayed in permeabilized myocytes under different conditions. Specifically, they found no differences in force development with and without phosphocreatine available. This finding was interpreted to reveal that “direct channeling of ATP and ADP between mitochondria and ATP-utilizing structures such as the SR and myofilaments establishes a direct crosstalk between organelles through compartmentation of adenine nucleotides” [49]. While the physical basis of the proposed “direct channel” is not clearly spelled out, an obvious candidate would be the well-defined physical chemical mechanism of molecular diffusion.

Evidence for a more crucial role of a CK-mediated transport system includes observations that the ADP concentration necessary to achieve the half-maximal rate of oxidative phosphorylation (*K*_ADP_) is approximately 0.015 mM in isolated cardiac mitochondria and 0.30 mM in suspensions of permeabilized cardiomyocytes [50],[51]. This difference can be explained by a severe restriction in the diffusivity of ADP within the cardiomyocyte, with an apparent diffusion coefficient more than one order of magnitude lower than in dilute solution. Indeed, it has been proposed that the effective diffusion coefficients of both ATP and ADP are restricted in cardiomyocytes [52]. If the diffusivities of ATP and ADP are as low as has been suggested by Saks and colleagues, then the conclusions of Meyer et al. [45] on a lack of significant gradients of phosphate metabolites on the micron scale in cardiomyocytes would not be valid. In that case, transport of ATP hydrolysis potential via the creatine–phosphocreatine system would be crucial for normal energy metabolism in the heart.

However, the picture painted by the permeabilized cell data is not entirely clear, because the permeabilized cell experiments lack an ideal control. Specifically, the apparent *K*_ADP_ in permeabilized fibers is not compared to the apparent *K*_ADP_ of mitochondria isolated from permeabilized cells. Thus arises one possible alternative explanation for the observed phenomenon: detergents used to permeabilize cells affect the mitochondrial membranes and consequently alter the apparent mitochondrial *K*_ADP_, perhaps by reducing the permeability to ADP and ATP or by depleting mitochondria of cytochrome c. In one study measuring *K*_ADP_ in permeabilized fibers and purified mitochondria [51], the detergent saponin was added to the mitochondrial respiration media in an apparent attempt to address this question. However, in that study, cell permeabilization required 30-minute incubation with saponin, while the isolated mitochondrial protocol involved measuring the respiration rate immediately following addition of the detergent at similar concentrations. Therefore the interpretation of these results is not as straightforward as has been suggested. (Additional results from experiments using digitonin to disrupt the outer membrane are discussed below in terms of subhypothesis 2.)

In addition, more direct observations on effective diffusion coefficients in striated muscle cells directly challenge subhypothesis 1. Kushmerick and Podolsky [43] showed by tracer methods that ATP diffusivity in skeletal muscle, measured in the direction of the long axis of a muscle fiber, is reduced only 2-fold relative to aqueous solution. Using an optical method, Vendelin and Birkedal [53] recently reported a similar result for ATP bound to a fluorescent dye. Perhaps more relevant to ATP transport from mitochondria to sites of ATP hydrolysis, they found an approximately 3-fold reduction in diffusivity in the plane perpendicular to myofibril orientation, compared to aqueous solution. Anisotropic diffusivity of ATP and PCr was measured by NMR methods in large goldfish muscle fibers, with axial diffusivity greater than radial [54],[55]. A similar result was found in lobster muscle [56]. The 3-fold reduction measured by Vendelin and Birkedal does not agree with the 20-fold or greater reduction predicted by Vendelin et al. [52] or the 1,000-fold reduction predicted by Selivanov et al. [57]. At the very least, the alternative hypothesis that the diffusivities of ATP and ADP are not restricted by more than a few fold in vivo compared to in dilute solutions lacks independent disproof and remains viable. Finally, there is the question of the apparent selectivity of the diffusion restrictions: theoretical formulations of the phosphocreatine shuttle hypothesis invoke restrictions of adenine nucleotide diffusivity, but not on the diffusivity of creatine or phosphocreatine [39],[52]. It is unclear what mechanism could be responsible for this selectivity beyond the approximately 2-fold difference in diffusivity based on molecular weights.

#### Subhypothesis 2: There is functional coupling between mitochondrial CK and ANT

Saks et al. [58] hypothesized that the mitochondrial ANT is functionally coupled to a creatine kinase isoform (mtCK) associated with the inner mitochondrial membrane. The specific hypothesis is that ATP translocated from the mitochondrial matrix can be directly transferred from the ANT to the active site of mtCK, where the reaction Cr+ATP→CrP+ADP is catalyzed [59]. The alternative hypothesis is that ATP must unbind from ANT and enter into aqueous solution before it is available for binding to mtCK. Thus subhypothesis 2 is that ANT and mtCK operate as a multi-enzyme complex with direct product–substrate channeling. The alternative hypothesis is that the transporters and the enzyme catalyze sequential steps in a pathway with independent catalytic mechanisms.

Saks et al. [59] present two lines of evidence in support of direct-transfer function coupling: “kinetic evidence” and “thermodynamic evidence.” Kinetic experiments, reproduced many times in the Jacobus and Saks laboratories [60]–[62], consistently show that the apparent maximal flux (*V*_max_) and Michaelis-Menten constant (*K*_m_) for ATP for the reaction Cr+ATP→CrP+ADP, assayed in suspensions of purified mitochondria, depend on whether or not there is a net flux through the ANT. Specifically, when mitochondria are synthesizing ATP, the apparent *V*_max_ is increased and the apparent *K*_m_ for ATP decreased, suggesting that mtCK turns over more readily when ATP is provided from ANT than when it is available only from the bulk solution. The thermodynamic evidence is that under certain conditions when Cr and ADP are available to respiring purified mitochondria, they are able to synthesize CrP in concentrations such that [CrP]·[ADP]/( [Cr]·[ATP]) exceeds the equilibrium mass-action ratio of the CK reaction Cr+ATP→CrP+ADP [61]. This phenomenon would be impossible unless the thermodynamic state in the compartment where the reaction is catalyzed were different from that of the bulk solution. A third line of evidence comes from tracer labeling experiments, where incorporation of labeled Pi into CrP in suspensions of respiring mitochondria shows preferential access of mtCK to ATP synthesized by oxidative phosphorylation versus ATP in the bulk solution [63],[64].

All of these observations may be explained by the direct-transfer hypothesis [59]. An alternative explanation is that there is a diffusion/permeation–limited region in the neighborhood of the mitochondrial inner membrane that offers some resistance to transport from the buffer solution to the mtCK. To test the diffusion barrier hypothesis, Erickson-Viitanen et al. [63] (in the Bessman laboratory) showed that when the outer mitochondrial membrane is permeabilized with digitonin, no functional coupling is apparent in either kinetic or tracer-labeling experiments. They concluded that the outer membrane provides a diffusion barrier that establishes a “microenvironment” that is responsible for mtCK-ANT coupling. Saks et al. [61] repeated the Erickson-Viitanen et al. kinetic experiments and observed the opposite result, that digitonin treatment did not affect the apparent functional coupling. They concluded that “the removal of the outer membrane does not alter the unique coupling between oxidative phosphorylation and mitochondrial creatine kinase,” and that the functional coupling “is the result of protein–protein proximity at the inner membrane surface.”

There is an obvious unresolved conflict when two labs report opposite results for the same experiment. Either one or both of the results is incorrect. In this case, neither result is entirely clear because it is unknown how oxidative phosphorylation could be viable with the “removal of the mitochondrial outer membrane” since cytochrome c—a necessary redox carrier in the electron transport system—diffuses freely in the intermembrane space. It is possible that the digitonin treatment in these experiments yielded a heterogeneous distribution of partial permeabilization and functional characteristics of mitochondria. Regardless of any possible explanation, from the conflicting results it can only be concluded that the hypothesis that the outer membrane presents a permeability barrier that is responsible for the apparent functional coupling remains without a rigorous attempt at disproof.

Finally, kinetic experiments on the purified mtCK enzyme [65] and mtCK reconstituted in liposomes [66] yield estimates of the *K*_m_ for ATP of approximately 56 µM and 48 µM, respectively. These values—lower than apparent *K*_m_ values estimated for nonrespiring mitochondria, which are in the range of 100 µM and higher [59]—are consistent with the permeability barrier hypothesis. In the spirit of strong inference, we construct the following hypothesis based on all of the above observations:

#### Subhypothesis 2.1: There is a permeability barrier associated with the mitochondrial outer membrane, and mtCK and ANT are not catalytically coupled through direct transfer of bound ATP

This is a simple, clear and unambiguous statement that is phenomenologically consistent with all of the independently reproduced data on functional coupling of mtCK and ANT. What remains is to determine if this statement can be successfully cast in terms of a quantitative hypothesis and associated theoretical/computational model. Attempts so far have not been successful. For example, Vendelin et al. [67] compared a kinetic model that accounted for a permeability barrier to one that invoked direct ATP channeling between ANT and mtCK and found that only the direct-channeling model could match the kinetic data from purified mitochondria. While this study did effectively disprove their permeability barrier model, the authors' conclusions that the permeability barrier hypothesis “is not sufficient to reproduce” the data and that “direct transfer is involved in the phenomenon of functional coupling” are not clearly justified. This is because Vendelin et al. convincingly demonstrated that one particular model that did not invoke direct-transfer cannot explain their data, not that all possible models cannot. To be certain, the disproved model in their study was perfectly reasonable and expertly constructed, providing strong support to, but not proof of, the direct-transfer hypothesis. Thus we have the alternative to the permeability-barrier hypothesis:

#### Subhypothesis 2.2: The catalytic mechanisms of mtCK and ANT are coupled in a way that allows for direct transfer of bound ATP between the active sites of ANT and mtCK

Saks and colleagues promote a hypothesis that “ATP is directly channeled by ANT from matrix into microcompartment (‘gap’) between” ANT and mtCK [68]. Because the physical nature of this microcompartment is not concretely defined, it is not clear if the view of Saks and his colleagues is consistent with subhypothesis 2.1, subhypothesis 2.2, or neither. Therefore we propose clearly disproving one or both of these simple alternatives before moving on to more complex ones. One possible experiment would be to make independent measures of ATP synthesis and of Cr and PCr transport over a range of fluxes and test the constancy of the stoichiometry. It might be possible to add CK to a reconstituted rotary ATP synthase motor for this purpose. The ultimate theory of mtCK and ANT kinetics may be complex, but to be useful (particularly as a computational model), it must be unambiguous and physically sound. To settle this issue, apply Platt's strategy by writing down clear alternative hypotheses. (Of course, it is possible and perhaps likely that neither hypothesis adequately represents reality.)

#### Subhypothesis 3: creatine kinase (non)equilibrium

The subhypothesis that the CK reaction mass-action ratio can be far from equilibrium in the cytoplasm of a cardiomyocyte is commonly proposed as a component of the phosphocreatine shuttle hypothesis [68]. Like subhypotheses 1 and 2, this subhypothesis is not necessary for the CrP/Cr-facilitated transport illustrated in Figure 3 when all metabolites are independently diffusing. In fact, while restricted diffusion of ADP and ATP and catalytic coupling of ANT and mtCK would tend to enhance the role of the CrP/Cr system in transporting ATP hydrolysis potential, the further away the CK reaction is from equilibrium, the smaller the facilitated transport flux [45].

Simulation studies of Saks and Aliev [69] and Vendelin et al. [39] have suggested that ADP concentration during cardiac contraction can rise several fold higher than would be predicted by CK equilibrium. However, these models made at least two assumptions that would tend to magnify the phenomenon: first, the activity of CK in a myofibrillar compartment was set relatively low (6 mmol·sec^−1^·(kg tissue)^−1^ versus values in the range of 25–40 mmol·sec^−1^·(kg tissue)^−1^ measured for the nonmitochondrial isoforms in vivo [70],[71]) and invoked the hypothesis of restricted diffusion of adenine nucleotides in the cytoplasm. Thus the phenomenon of the CK reaction operating far from equilibrium is a designed feature of these simulations rather than a prediction. Second, a factor was build into the transport equations, specifically reducing the permeability of the mitochondrial membrane to ADP, as discussed previously. We point these details out not to argue that these features are necessarily invalid in myocytes, but only to clarify a key difference between a model assumption and a model prediction. These particular model assumptions do not stand on independent experimental justification and like the other hypotheses and subhypotheses outlined above, requires a strong-inference–based investigation to sort out.

## Conclusions and Recommendations

Here we have demonstrated the utility of applying Platt's strong inference to critical questions in cardiac energy metabolism. Regarding the hypothesis of metabolic stability, Platt's rigorous logic provides a framework for disproof and for establishing and testing alternatives. In doing so, we have shown seemingly contradictory data (on metabolic kinetics in purified mitochondria [28],[30] and phosphate metabolites in the heart in vivo [29],[31]) may be synthesized into a coherent theory. Regarding the phosphocreatine shuttle, strong inference reminds us that before disproof may even be attempted the theory must be posed via Platt's first step of laying out clear alternative hypotheses. Toward that goal, we have cast the phosphocreatine shuttle hypothesis as a set of hypotheses to be tackled using computational biology as the vehicle for applying strong inference. For both of these examples, computational modeling has and will be applied as a key tool for formulating explicit hypotheses and in designing experiments with sufficient power to disprove a hypothesis. This is because the systems under investigation are so complex that hypothetical biochemical mechanisms can be realistically represented only through simulation.

Hence further progress in this field, and in biological systems research in general, will rely on further application of strong inference. Likewise, application of strong inference to complex biological systems requires computational simulation to formulate the hypotheses, to compare hypotheses to data, and to design the experiments to distinguish between alternatives. Biological systems research, strong inference, and computational modeling are constructively and inseparably coupled.
